# High-resolution proteomics reveals differences in the proteome of spelt and bread wheat flour representing targets for research on wheat sensitivities

**DOI:** 10.1038/s41598-020-71712-5

**Published:** 2020-09-07

**Authors:** Muhammad Afzal, Jens Pfannstiel, Julia Zimmermann, Stephan C. Bischoff, Tobias Würschum, C. Friedrich H. Longin

**Affiliations:** 1 State Plant Breeding Institute, University of Hohenheim, Fruwirthstrasse 21, 70599 Stuttgart, Germany; 2 Core Facility Hohenheim, University of Hohenheim, August-von-Hartmann-Strasse 3, 70599 Stuttgart, Germany; 3 Institute of Nutritional Medicine, University of Hohenheim, Fruwirthstrasse 12, 70599 Stuttgart, Germany; 4 Institute of Plant Breeding, Seed Science and Population Genetics, University of Hohenheim, Fruwirthstrasse 21, 70599 Stuttgart, Germany

**Keywords:** Immunology, Molecular biology, Plant sciences

## Abstract

Wheat consumption can trigger celiac disease, allergic reactions and non-celiac wheat sensitivity (NCWS) in humans. Some people with NCWS symptoms claim a better tolerability of spelt compared to bread wheat products. We therefore investigated potential differences in the proteomes of spelt and bread wheat flour using nano LC–ESI–MS/MS on a set of 15 representative varieties for each of the two species. Based on the bread wheat reference, we detected 3,050 proteins in total and for most of them the expression was mainly affected by the environment. By contrast, 274 and 409 proteins in spelt and bread wheat, respectively, had a heritability ≥ 0.4 highlighting the potential to influence their expression level by varietal choice. We found 84 and 193 unique proteins for spelt and bread wheat, respectively, and 396 joint proteins, which expression differed significantly (*p* ≤ 0.05) when comparing both species. Thus, about one third of proteins differed significantly between spelt and bread wheat. Of them, we identified 81 proteins with high heritability, which therefore might be interesting candidates for future research on wheat hypersensitivities.

## Introduction

The consumption of bread wheat (*Triticum aestivum* ssp. *aestivum*) products can cause different diseases in humans like celiac disease (CD), allergic reactions and non-celiac wheat sensitivity (NCWS) which might affect up to 10% of the human population^1,2^. Spelt (*Triticum aestivum* ssp. *spelta*) is a wheat species defined as a different subspecies to bread wheat. Both spelt and bread wheat are hexaploid having the AABBDD genome and are able to naturally cross with each other delivering fertile progenies. Despite these similarities, millers and bakers selling both spelt and bread wheat products were confronted with consumers claiming to have health problems ranging from symptoms like flatulence to diseases like neurodermatitis when eating bread wheat but not when eating spelt products.

Although several studies compared spelt with bread wheat, a clear scientific proof of this phenomenon based on robust human studies using well-defined raw material is lacking^3,4^. Comparison of different ingredients like gluten composition^5^ and lipophilic antioxidants^6^ showed differences between spelt and bread wheat, but also a considerable variation within both species, which could even be larger than across species^4^. However, using 5,061 neutral molecular markers (SNPs), Akel et al.^7^ showed that 198 spelt varieties clustered genetically separate to 155 elite bread wheat varieties in a principal coordinate analysis. Similarly, considerable divergence between genome sequence of spelt and bread wheat have been reported^8^. Consequently, more research is required to identify different ingredients in spelt and bread wheat based on well-defined raw material^4^.

The known epitopes for CD and wheat allergy are proteins^2^. For NCWS, the research and development of diagnostics are yet ongoing, and several hypotheses exist on potential triggers, which also include proteins like α-amylase trypsin inhibitors (ATIs)^9^. Serpins can trigger allergic reactions^10^ and CD^2^ whereas wheat germ agglutinin (WGA) is reported to cause CD^2^. Therefore, a detailed characterization of differences in spelt and bread wheat proteomes is essential to identify proteins potentially involved in NCWS and other diseases caused by the consumption of wheat products. Within the last decade striking advances were made in mass spectrometry technology^11^ that allowed the in-depth analysis of whole proteomes ranging from lower eukaryotes^12^ to humans^13^ and plants^14^. The recent publication of an annotated wheat genome sequence^15^ now enables a detailed characterization of wheat at the proteome level using modern mass spectrometry methods. However, this technology has, to the best of our knowledge, not been extensively used to investigate the proteomes of spelt and bread wheat.

We therefore used 15 varieties representative for the current production of spelt and bread wheat in Germany, which were grown in three different test locations and analysed their flour proteome using Nano LC–ESI–MS/MS. Our objectives were to (1) investigate whether spelt and bread wheat flour contain different proteins, which might be further targets for research on NCWS, (2) compare the between- and within-species variation, and (3) elaborate the impact of environment versus genetics on the expression of proteins in order to discuss possible consequences for the future wheat supply chain as well as human and animal nutrition.

## Results and discussion

**Figure 1:**
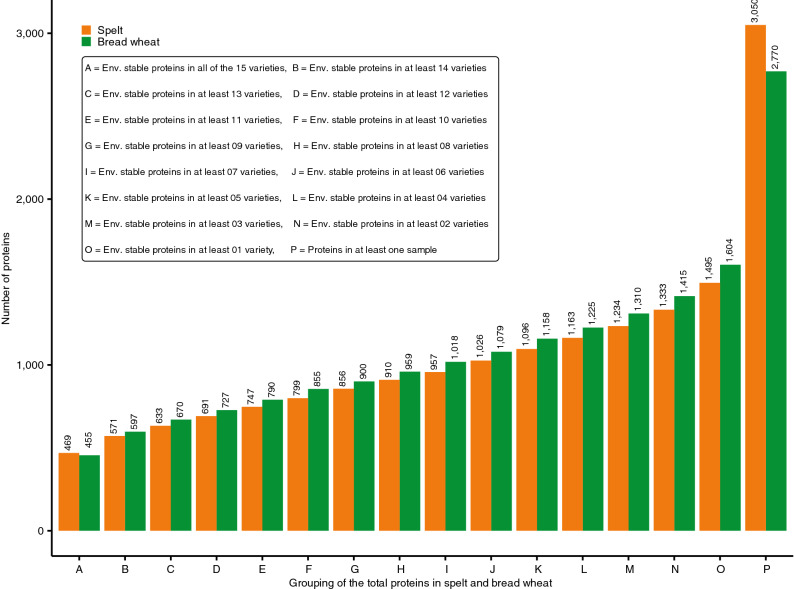
Grouping of the proteins identified in spelt and bread wheat. The y-axis shows the number of proteins for each group. On the x-axis are groups assigned based on the proteins expressed in at least one sample (group P) or the proteins which were consistently identified at all environments in a certain number of varieties i.e. 1–15 (*Env.* environmental).

In our proteomics study we were able to detect 3,050 and 2,770 proteins in total in spelt and bread wheat, respectively (Fig. 1), which is to the best of our knowledge the largest yet reported number for spelt. Interestingly, we were able to identify even more proteins in spelt samples than in bread wheat samples although we had to use the wheat reference protein sequence also for spelt as no published spelt reference exists. A positive mass spectrometry identification of peptides from spelt proteins requires sequence identity to the corresponding bread wheat peptides, indicating that protein sequences are highly conserved between spelt and bread wheat. Whether this holds true also for other wheat subspecies requires further research. Out of the identified proteins, approximately 60% were annotated with a name in the wheat reference database (UniProt). Numerous protein names are inferred from electronical annotation based on typical protein sequence motifs (cf. “AAI-rich protein”) and lack experimental verification of the proposed function. That underlines that state-of-the-art proteomics tools open fully new ways to the analyses of whole proteomes with quite fast processing time and high sample throughput. In parallel, studies are required to deepen our knowledge on protein functions and to establish reference sequences for each species.

### Protein expression largely driven by environmental effects

From the total number of identified proteins, 1,555 proteins in spelt and 1,166 in bread wheat were only detected in a subset of the field locations (Fig. 1). Thus, their expression could be completely repressed depending on the specific environmental conditions at the growing locations and we were unable to identify a specific trend showing for instance that most proteins were expressed at one location and the fewest at another location. It rather appears that these proteins were expressed in an environment-specific manner, underlining a large environmental impact on protein expression. We therefore focused our further investigations on those proteins, which were consistently expressed across all test locations in at least one variety within a species. Those were 1,495 and 1,604 proteins in spelt and bread wheat, respectively.

**Figure 2:**
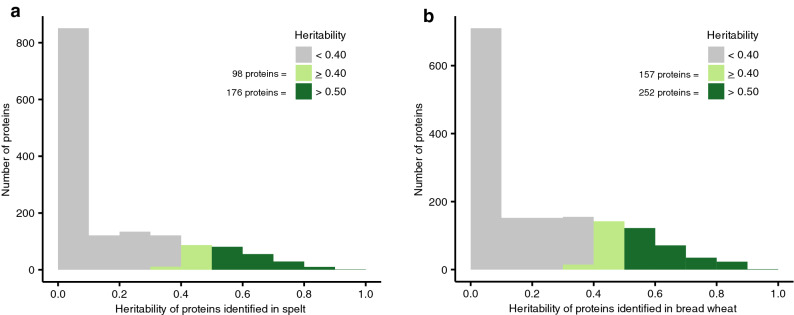
Histogram of the heritability values of all proteins with environmentally stable expression in at least one variety (i.e. group O in Fig. 1) in (**a**) spelt and (**b**) bread wheat.

Even for those proteins, a large impact of the environment on their level of expression was visible (Fig. 2). The heritability of a trait determines to which extent the visible variation in the field (i.e. the expression level of a protein) is influenced by genetic versus environmental factors. It ranges between 0 (no genetic influence, only environmental influence) and 1 (only genetic influence) and varies for important agronomic traits like grain yield or kernel raw protein content in wheat between 0.5 and 0.8^16,17^. For the proteins, which were environmentally stable expressed in at least one variety, 81.7% in spelt and 74.5% in bread wheat had a heritability value < 0.4 indicating a very large environmental impact on their expression. By contrast, only 11.7% of these proteins in spelt and 15.7% in bread wheat had a high heritability value > 0.5. These findings are in line with data on the classically determined kernel raw protein content of wheat grains, which is determined for instance by Dumas combustion principle (ICC standard method 167, ICC, Vienna, Austria). This kernel raw protein content is largely affected by environmental conditions including the amount and type of nitrogen fertilization, the weather conditions and soil types influencing the availability of nutrients for the plant^18,19^.

On the one hand, these results show a large environmental influence on protein expression, thus suggesting careful choice of sample material for scientific research purposes. For instance, if the amount of a certain protein shall be quantified in a variety or if different varieties within a species shall be compared, samples grown next to each other at different field locations have to be analysed in order to be able to separate the environmental effect from that of the variety^4^. On the other hand, all proteins and other components of samples, which presence is mainly affected by environmental factors, are impossible to be controlled or specifically manipulated in regular agricultural supply chains. A farmer can, for sure, try to standardize the fields and field treatments in crop production, but the climate is different every year leading to different availabilities of water and nutrients from the soil. The importance of this effect could be seen from the analysis of variance (ANOVA) in official variety trials, where the same varieties were grown in several field locations across different years with the prerequisite of a similar field management by the respective farmers. Based on that specific field design, the total visible variance can be separated into variances caused by the varieties but also in variances arising from the cropping year, the test location, the variety-by-location, variety-by-year and variety-by-location-by-year effects. Interestingly, the size of the latter components is often as large or even larger than the variety variance in numerous crops^20,21^ underlining the large impact of the environment on the expression of traits. Therefore, only those proteins which are environmentally stable expressed and thus have a moderate to high heritability, can be controlled across supply chains for instance by choosing a variety which has a low expression of a protein with potential negative health effects for humans (cf. Fig. 6).

### Varietal choice influences protein expression in supply chain

**Figure 3:**
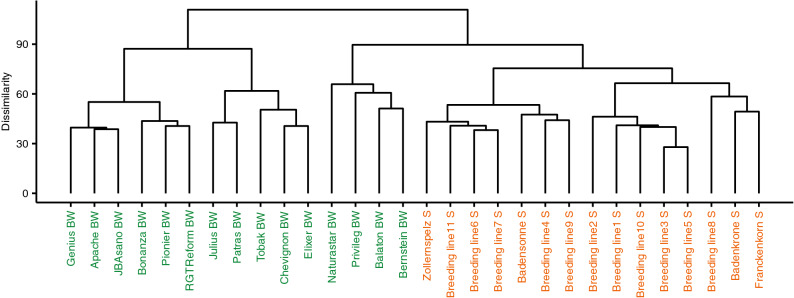
Hierarchical clustering of the spelt and bread wheat varieties based on all proteins, which were environmentally stable expressed in at least one variety (i.e. group O in Fig. 1; *BW *bread wheat, *S* spelt).

**Figure 4:**
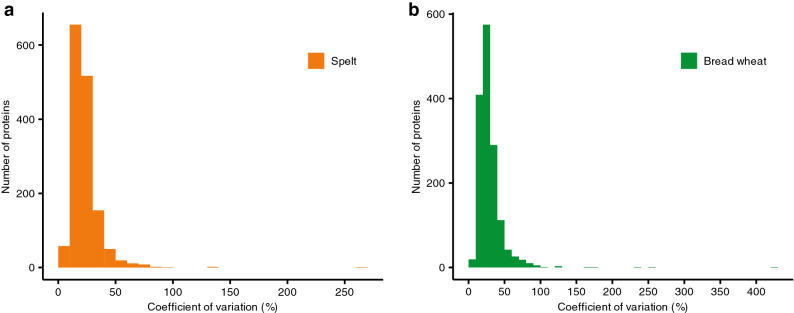
Coefficient of variation of proteins, which were environmentally stable expressed in at least one variety (i.e. group O in Fig. 1) for (**a**) spelt and (**b**) bread wheat.

In spelt and bread wheat, the investigated varieties differed largely in their expression of the investigated proteins (Figs. 1, 3, 4, 6, Supplementary Tables S1–S3 online). First, this was observed as an environmentally stable present/absent variation between different varieties. For instance, only 469 and 455 proteins were expressed in all 15 varieties of spelt and bread wheat, respectively (Fig. 1). By contrast, up to 1,495 in spelt and 1,604 proteins in bread wheat were expressed environmental stable in at least one variety. Thus, two thirds of environmentally stable expressed proteins were present only in some but not in all varieties. Second, for proteins expressed environmental stable in several varieties, we observed a considerable variation in their expression level between different varieties with a coefficient of variation following roughly a normal distribution for the different proteins with a mean of 22.85% and 29.06% for spelt and bread wheat, respectively (Fig. 4).

For those proteins, where a large genetic variance is coupled with a moderate—high heritability, the choice of certain varieties can be a powerful method to successfully manipulate their amount along agricultural supply chains until the consumers. Taking a heritability value of 0.4 as threshold in our analyses, this would apply for 274 and 409 proteins in spelt (Supplementary Table S1 online) and bread wheat (Supplementary Table S2 online), respectively, comprising beside numerous uncharacterized proteins also few alpha-amylase inhibitors, serpins, gliadins, glutenins and one Bowman-Birk trypsin inhibitor. In future, targeted plant breeding could even increase or decrease the amount of these proteins, if the flour and bread market would ask and pay for wheat varieties with specific protein portfolios. These findings are similar to the results regarding other wheat-grain ingredients, for instance lipophilic antioxidants^6^, mineral content^22^ or sulfur and asparagine content^23^. Consequently, analyses of ingredients in plant products requires not only accurate sampling strategies across different test environments but also the composition of a representative set of varieties for the investigated crop. For further investigation of the genetic architecture of the investigated proteins, a high-density genotyping coupled with the analysis of proteins in > 150 different varieties per species grown at several field locations would be necessary, warranting further research.

### Comparing spelt with bread wheat

Spelt and bread wheat belong to the same species but are different subspecies. They have both the AABBDD genome and can naturally cross with each other. Despite these similarities, millers and bakers selling both spelt and bread wheat products are often confronted with consumers who claim to have health problems when eating bread wheat products but not when eating spelt products. To compare spelt and bread wheat, we generated a dendrogram using hierarchical clustering of the 15 spelt and 15 bread wheat varieties based on all proteins which were environmentally stable expressed in at least one variety (Fig. 3). We identified two major groups. The first group comprised of eleven bread wheat varieties and the second group of four bread wheat and all spelt varieties. Nevertheless, within the latter group, a clear separation was observed between four bread wheat and 15 spelt wheat varieties. This underlines the large variability within species already discussed above, but also shows that the variation between the two species appears to be even larger. This confirms two genomic studies comparing spelt and bread wheat based on two different genotyping approaches^7,8^.

**Figure 5:**
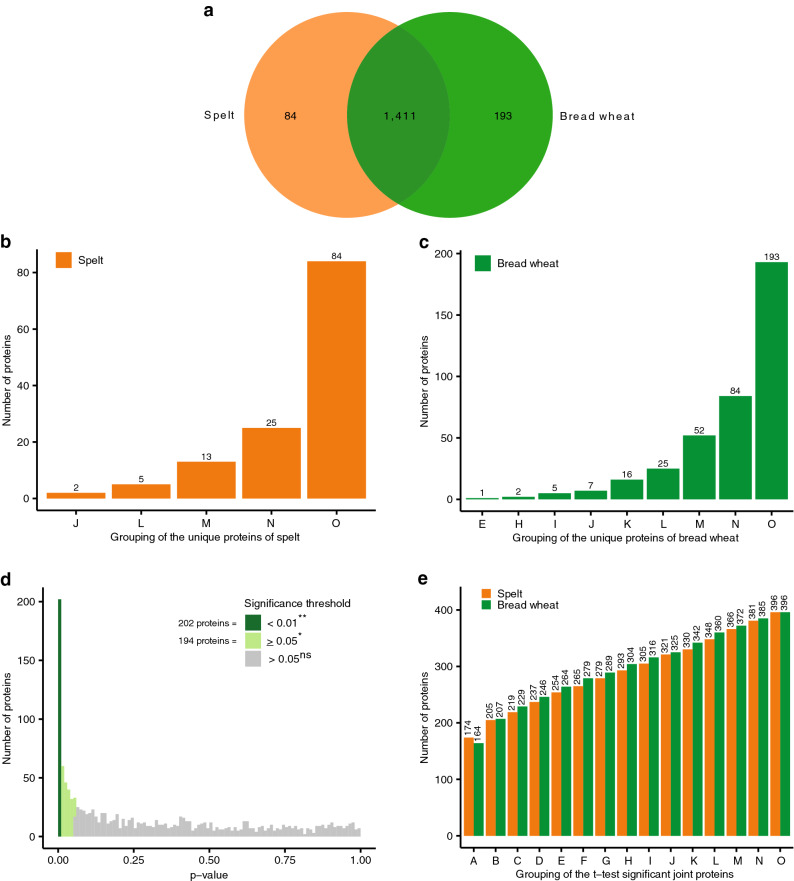
Comparing spelt with bread wheat. (**a**) Numbers of proteins expressed environmentally stable in at least one variety, which were either unique to spelt or bread wheat or jointly expressed in both species; (**b**) Grouping of the unique proteins for spelt, which were environmentally stable expressed in at least some varieties; (**c**) Grouping of the unique proteins for bread wheat, which were environmentally stable expressed in at least some varieties; (**d**) Histogram of the *p* values from the *t* test of the jointly expressed proteins in spelt and bread wheat, which were environmentally stable expressed in at least one variety in spelt and bread wheat (i.e. group O in Fig. 1); (**e**) Grouping of the joint proteins between spelt and bread wheat, which were environmentally stable expressed in at least some varieties as well as significantly differentially expressed (*p* ≤ 0.05) in the two species according to the *t* test (for letters on x-axis in (**e**), see Fig. 1).

Furthermore, we identified 84 and 193 proteins which were unique for spelt and bread wheat, respectively (Fig. 5a). As no reference proteome of spelt exists, we had to use the bread wheat proteome for identification of spelt proteins. Therefore, the sequence of peptides from spelt proteins must be identical to their wheat counterparts to enable identification of spelt proteins by mass spectrometry. Missing the identification of proteins in spelt, which were present in bread wheat, could either be due to the fact that they were absent in spelt or that the spelt protein was slightly different than the bread wheat protein. Thus, for the 193 unique proteins in bread wheat, which were not detected in spelt, we cannot rule out that all these proteins do exist in spelt. In contrast, the 84 proteins, which were not detected in bread wheat but in spelt, were unique proteins identified in spelt varieties analysed in this study. With the availability of a spelt reference genome in the future, the list of proteins unique for spelt might become even longer. However, many of the identified unique proteins were present only in a few varieties minimizing their importance across the agricultural supply chain (Fig. 5b, c).

A fairly large number of environmentally stable expressed proteins, i.e. 1,411, were detected in both spelt and bread wheat varieties. Interestingly, 396 of these 1,411 joint proteins had a significantly different expression level (*p* ≤ 0.05) between spelt and bread wheat as determined by a *t* test (Fig. 5d). Thereof, 265 and 279 proteins were present in at least ten varieties in spelt and bread wheat, respectively (Fig. 5e). The presence of these proteins in a high number of varieties means that they were also widely grown by farmers and, thus, also widely consumed by human and animal consumers via spelt and bread wheat products. Altogether, comparing the sum of the significantly differentially (*p* ≤ 0.05) expressed and unique proteins to those proteins, which were environmentally stable expressed in at least one variety (i.e. group O in Fig. 1), about 32% and 37% of proteins in spelt and bread wheat, respectively, differed statistically significantly between the two species.

**Figure 6:**
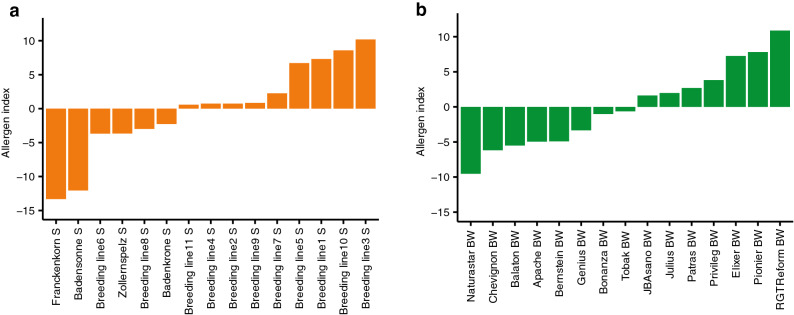
Allergen index for all tested varieties of (**a**) spelt (*S* spelt) and (**b**) bread wheat (*BW* bread wheat).

Proteins are main epitopes in CD and wheat allergy and are also discussed as potential triggers for wheat sensitivity^2^. For example, ATIs trigger bakers’ asthma and are also discussed as potential causes for NCWS^9^. Serpins can cause wheat allergy^10^ and CD^2^, whereas WGA has been reported to cause CD^2^. Based on all proteins identified in our dataset that were annotated as ATIs, serpins and WGA (Table 1), we calculated an “allergen index” for all tested varieties. As the LFQ values differed largely between various proteins, we first standardized the LFQ values of all proteins and then summed them up separately for each variety, i.e. built up an equally weighted index. We observed a large variation across the 15 varieties within spelt and bread wheat ranging from − 13.32 to 10.88 (Fig. 6). In spelt, the most popular varieties Franckenkorn, Zollernspelz and Badensonne had relatively low values of the allergen index. In the commonly used European commercial bread wheat cultivars, there were contrasting values, with Chevignon having a low and RGTReform a high allergen index. These findings underline the possibility to control protein amounts and protein portfolio of wheat products via choice of varieties at the beginning of the wheat supply chain. This is of particular interest, because currently spelt and bread wheat varieties are only judged along the supply chain by farmers, millers and bakers based on their agronomic performance and baking quality but not at all on their ingredients. Nevertheless, our allergen index represents just one beyond several other simple methods for combining traits in order to illustrate the variability between different varieties in the expression of potential allergenic proteins. Much more research is required on the proteins expressed in cereals, their functionality in grains and plants, their role in human/animal nutrition, and—most importantly—in hypersensitivity reactions.

**Table 1:** Details about proteins that were used to calculate allergen index for each variety of spelt and bread wheat.

Protein no.	Majority protein IDs	Shortened protein annotations (UniProt)	Mol. weight (kDa)	Spelt		Bread wheat	
				H^2^	No of varieties^a^	H^2^	No of varieties^a^
prot3912	P01085; A0A3B6GR96; A0A3B6GNC7	sp|P01085|IAA1_WHEAT Alpha-amylase inhibitor 0.19	13.337	0.51	15	0	15
prot4185	X2KYP9; P01083	sp|P01083|IAA2_WHEAT Alpha-amylase inhibitor 0.28	16.628	0.64	15	0.36	15
prot3911	P01084	sp|P01084|IAA5_WHEAT Alpha-amylase inhibitor 0.53	13.185	0	14	0	14
prot3703	A0A3B6TFZ6; P16850	sp|P16850|IAAC1_WHEAT Alpha-amylase/trypsin inhibitor CM1	15.527	0.26	15	0	15
prot3939	P16851	sp|P16851|IAAC2_WHEAT Alpha-amylase/trypsin inhibitor CM2	15.460	0.21	15	0.14	15
prot3940	P17314	sp|P17314|IAAC3_WHEAT Alpha-amylase/trypsin inhibitor CM3	18.221	0.48	15	0	15
prot3936	P16159	sp|P16159|IAC16_WHEAT Alpha-amylase/trypsin inhibitor CM16	15.782	0	15	0.28	15
prot3985	Q4U1A4	tr|Q4U1A4|Q4U1A4_WHEAT Dimeric alpha-amylase inhibitor	15.046	0.55	15	0.15	15
prot3938	P16347; A0A3B6C9V8	sp|P16347|IAAS_WHEAT Endogenous alpha-amylase/subtilisin inhibitor	19.633	0.75	15	0.88	15
prot3932	P10846	sp|P10846|IAA3_WHEAT Alpha-amylase inhibitor WDAI-3	4.797	–	–	0.12	7
prot3981	Q41593; A0A3B6LS85	sp|Q41593|SPZ1A_WHEAT Serpin-Z1A	43.118	0.44	15	0.34	15
prot4033	Q9ST57	sp|Q9ST57|SPZ2A_WHEAT Serpin-Z2A	43.311	–	–	0.28	11
prot4034	Q9ST58	sp|Q9ST58|SPZ1C_WHEAT Serpin-Z1C	42.881	0.32	15	0.48	15
prot3885	H9AXB3	tr|H9AXB3|H9AXB3_WHEAT Serpin-N3.2	42.996	0.34	15	0	15
prot1967	A0A3B6HVL4	tr|A0A3B6HVL4|A0A3B6HVL4_WHEAT SERPIN	42.633	0	15	0.42	15
prot2139	A0A3B6IPZ0	tr|A0A3B6IPZ0|A0A3B6IPZ0_WHEAT SERPIN	42.582	0	9	0	9
prot2243	A0A3B6JDS9	tr|A0A3B6JDS9|A0A3B6JDS9_WHEAT SERPIN	42.617	0	1	0	2
prot2583	A0A3B6KQL2	tr|A0A3B6KQL2|A0A3B6KQL2_WHEAT SERPIN	43.152	0	15	0.11	15
prot2616	A0A3B6KSZ6	tr|A0A3B6KSZ6|A0A3B6KSZ6_WHEAT SERPIN	26.102	–	–	0	3
prot2853	A0A3B6MWJ8; P93693	tr|A0A3B6MWJ8|A0A3B6MWJ8_WHEAT SERPIN	43.070	0.04	15	0.19	15
prot3735	A0A3B6TLW2	tr|A0A3B6TLW2|A0A3B6TLW2_WHEAT SERPIN	43.417	0.4	15	0.73	15
prot0800	P10969; A0A3B5ZYD0; P10968; A0A3B5Z2G8; P02876	sp|P10969|AGI3_WHEAT Agglutinin isolectin 3	18.756	0	7	0.21	5

^a^The number of varieties in which the respective protein was expressed at all environments; H^2^ = Heritability; UniProt = universal protein knowledgebase, the hyphen “–” denotes the absence of a protein in spelt.

Our data can serve as a starting point for future research. For instance, we identified 396 proteins, which were environmentally stable expressed in spelt and bread wheat, but at statistically different amounts. In addition to these 396 proteins, we identified unique proteins for both species. Out of all these proteins, we selected 81 proteins which additionally had a heritability ≥ 0.4 in both species, thus have mainly been affected by the genetics of the varieties and to a lesser extent by the environmental conditions and can therefore be manipulated along the supply chain (Supplementary Table S3 online). Interestingly, beside many uncharacterized proteins only one serpin, one alpha amylase inhibitor (AAI), one Bowman-Birk type trypsin inhibitor and one xylanase inhibitor are on that list. The expression of three of them (AAI, Bowman-Birk type trypsin inhibitor and xylanase inhibitor) was significantly lower (*p* ≤ 0.05) in spelt than in bread wheat whereas the serpin had a significantly higher (*p* ≤ 0.05) expression in spelt than in bread wheat. To our opinion, these 81 proteins represent interesting candidates for future research on mechanisms of wheat hypersensitivity.

## Conclusions

By using advanced proteomics methods, high numbers of proteins can be detected in cereal grains paving new ways for research and applications in the wheat supply chain. We could clearly show that the expression of many proteins was mainly influenced by environmental conditions under which the varieties were grown. Considering variable climatic conditions in cereal production, only those proteins which are largely independent from environmental effects and instead primarily controlled by the grain genetics can effectively be manipulated across the wheat supply chain. We identified among several thousand proteins a few hundreds of such proteins in spelt and bread wheat. These proteins could be the targets in future plant breeding for optimization of the varietal characteristics according to the market demands. We further could show that approximately one third of the detected proteins differed between spelt and bread wheat. Moreover, concentrating only on proteins with high heritability, we presented a list of interesting candidate proteins for further research on wheat sensitivity.

## Methods

### Plant material and field trials

We investigated 15 different varieties of spelt (*Triticum aestivum* ssp. *spelta*) and bread wheat (*Triticum aestivum* ssp. *aestivum*) representative of the recent market diversity in Central Europe. For spelt, we used the four most important varieties of the market Badenkrone, Badensonne, Franckenkorn and Zollernspelz as well as eleven breeding lines of our spelt breeding program. For bread wheat, following varieties were used: Apache, Balaton, Bernstein, Bonanza, Chevignon, Elixer, Genius, JBAsano, Julius, Naturastar, Patras, Privileg, RGTReform and Tobak.

The field trials were conducted as winter cropping, i.e. sowing in October 2016 and harvest in July 2017, at three diverse locations in Germany and France. The two wheat species were investigated in separate trials at each location using an un-replicated field design randomized separately for each species across test locations. All trials received the same field treatments of intensive conventional farmer’s practice except for nitrogen fertilization, which was reduced by 50 kg/ha for spelt compared to bread wheat like common in agricultural practice. Field net plot size was 5 m^2^ in all locations. All plots were machine-sown and combine-harvested. All samples of spelt were dehulled and cleaned using a Mini-Petkus seed cleaner (Röber, Bad Oeynhausen, Germany) to separate hulls, straw and damaged kernels. Dehulling was performed using a classical stone mill, in which the stone was replaced by hard rubber. For bread wheat, seed cleaning was also performed using the Mini-Petkus seed cleaner in order to remove chaff and straw particles, which were still present after combine harvesting.

### Laboratory analyses

#### Protein extraction for comparative proteome analysis

We performed proteome analyses at the whole grain flour of the harvested samples, which was generated using Cyclotec mill (Foss GmbH, Germany). Twenty milligram (20 mg) spelt or bread wheat flour were suspended in lysis buffer containing 2% SDS (sodium dodecyl sulfate), 20 mM DTT (dithiothreitol) and 150 mM Tris–HCl pH 8.5 and incubated for 10 min at 95 °C. After centrifugation at 4 °C for 30 min at 13,700 rpm the supernatant was removed, and proteins were precipitated using chloroform–methanol precipitation^24^. Protein pellets were resuspended in 6 M urea in 50 mM Tris–HCl pH 8.5, and protein concentrations were determined by the Bradford assay^25^.

#### In-solution digest of proteins and peptide purification

Ten microgram (10 µg) spelt or bread wheat protein extract in 60 µl 6 M urea, 50 mM Tris HCl (pH 8.5) were used for in solution digests. DTT was added to a final concentration of 10 mM for the reduction of cysteines. Samples were incubated for 30 min at 56 °C under shaking at 1,000 rpm in an Eppendorf Thermomixer. Alkylation of cysteines was performed by adding 30 mM iodoacetamide and incubation for 45 min at room temperature (RT) in the dark. Alkylation was stopped by adding 50 mM DTT and samples were incubated for another 10 min at RT. Five hundred nanogram (500 ng) LysC protease (Roche) in 50 mM Tris HCl pH 8.5 was added and samples were digested overnight at 30 °C. Next, the urea in the reaction mixture was diluted to 2 M by adding the appropriate amount of 50 mM Tris HCl pH 8.5. One microgram (1 µg) trypsin (Roche) in 50 mM Tris HCl pH 8.5 was added and digestion was continued for 4 h at 37 °C. The digest was stopped by adding 3 µl 10% TFA (trifluoroacetic acid). Next, peptide mixtures were concentrated and desalted on C18 stage tips^26^ and dried under vacuum. Dried samples were dissolved in 30 µl 0.1% TFA. Aliquots of 3 µl were subjected to nanoLC-MS/MS analysis.

#### Mass spectrometry analysis

Nano LC–ESI–MS/MS experiments were performed on an UltiMate 3000 RSLCnano system (Thermo Fisher Scientific) coupled to a Q-Exactive HF-X mass spectrometer (Thermo Fisher Scientific) using a Nanospray Flex ion source (Thermo Fisher Scientific). Tryptic digests were concentrated and desalted on a precolumn (300 µm × 5 mm, Acclaim PepMap100 C18, 5 µm particle size, 100 Å pore size) and separated on a nanoEase MZ HSS T3 analytical column (25 cm × 75 μm, 1.8 µm particle size, 100 Å pore size, Waters) operated at constant temperature of 35 °C. Peptides were separated at a flow rate of 300 nL/min using a 90 min gradient with the following profile: 2%—55% solvent B in 90 min, 55%—95% solvent B in 5 min and maintained at 90% solvent B for 5 min. We used 0.5% acetic acid (solvent A) and 0.5% acetic acid in acetonitrile/H2O (80/20, v/v, solvent B) as solvents.

The Q Exactive HF-X was operated under the control of XCalibur 4.3.73 software. MS spectra (m/z = 300–1,800) were detected in the Orbitrap at a resolution of 60,000 (at m/z = 200) using a maximum injection time (MIT) of 100 ms and an automatic gain control (AGC) value of 1 × 10E6. Internal calibration of the Orbitrap analyser was performed using lock-mass ions from ambient air as described in Olsen et al.^27^. Data dependent MS/MS spectra were generated for the 30 most abundant peptide precursors in the Orbitrap using higher-energy C-trap dissociation (HCD) fragmentation at a resolution of 15,000, a normalized collision energy of 27 and an intensity threshold of 1.6×10E5. Only ions with charge states from + 2 to + 5 were selected for fragmentation using an isolation width of 1.6 Da. For each MS/MS scan, the AGC was set at 2×10E5 and the MIT was 50 ms. Fragmented precursor ions were dynamically excluded for 30 s within a 5 ppm mass window to avoid repeated fragmentation.

#### Protein quantification and data analysis

Raw files were imported into MaxQuant^28^ version 1.6.0.1 for protein identification and label-free quantification (LFQ) of proteins. Protein identification in MaxQuant was performed using the database search engine Andromeda^29^. MS spectra and MS/MS spectra were searched against wheat proteome sequence database downloaded from UniProt^30^. As no spelt reference genome is published yet, we blasted spelt also against the bread wheat reference. Reversed sequences as decoy database and common contaminant sequences were added automatically by MaxQuant. Mass tolerances of 4.5 ppm (parts per million) for MS spectra and 20 ppm for MS/MS spectra were used. Trypsin was specified as enzyme and three missed cleavages were allowed. Carbamidomethylation of cysteines was set as a fixed modification and protein N-terminal acetylation and oxidation were allowed as variable modifications. The ‘match between runs’ feature of MaxQuant was enabled with a match time window of one minute and an alignment time window of 20 min. Peptide false discovery rate (FDR) and protein FDR thresholds were set to 0.01. The mass spectrometry proteomics data will be deposited to the ProteomeXchange Consortium via the PRIDE partner repository with the dataset identifier.

### Phenotypic data analysis

Phenotypic data analysis was performed according to the linear mixed model, given in Eq. (1):

1 $$y_{ik} = u + v_{i} + env_{k} + e_{ik} ,$$

where *y*_ik_ was the phenotypic observation for the *i*th variety tested in the *k*th environment, *u* was the general mean, *v*_i_ the varietal effect of the *i*th variety, *env*_k_ the effect of the *k*th environment, and *e*_ik_ was the residual error.

Variance components, which were variance due to varieties, environments and residual error, were estimated using the restricted maximum likelihood (REML) method assuming a random model in a classical one-stage analysis^31^. These variance components are shown in Supplementary Table S4 and S5 online. A likelihood ratio test with model comparisons was performed^32^ to check for significance of the variance components. Average values of the proteins across the different environments were determined as best linear unbiased estimates (BLUEs) assuming fixed genetic (variety) effects. Heritability estimates (${h}^{2}$) were computed following Piepho and Möhring, 2007^33^ as given in Eq. (2):

2 $$h^{2} = 1 - \frac{\vartheta }{{2\sigma_{G}^{2} }},$$

where ϑ is the mean variance of a difference of two best linear unbiased predictors and $\sigma_{G}^{2}$ the genotypic variance (varietal variance). All analyses were performed utilizing the statistical software R^34^ and the software ASReml 3.0^35^.

#### Student’s t test

As only the two groups spelt and bread wheat were compared, we used a *t* test and not an ANOVA. We applied an independent-samples Student’s *t* test (α = 0.05)^36^ to compare the expression level of a protein between spelt and bread wheat. For the *t* test, the BLUEs of each protein for each variety of spelt and bread wheat were used. The statistical model (during phenotypic analysis) did not converge for some of the joint proteins. Those proteins (11 in spelt, 24 in bread wheat) had, on average, 82% and 76% missing values across 15 varieties each of spelt and bread wheat, respectively. Therefore, the *t* test was not conducted for 35 joint proteins. Hence, the number of the joint proteins for the *t* test was reduced from 1,411 to 1,376. For independent two samples *t* test, the assumption of equality of variances was examined by applying Levene's test^37^. If Levene’s test was significant (*p* < 0.05, meaning that the variances are not equal), then the more robust Welch's *t* test was conducted instead of the regular independent samples *t* test, with corrected degrees of freedom reported to two decimal places. Student’s *t* test and Levene’s test were implemented using the statistical software R^34^.

#### Calculation of allergen index

To calculate the allergen index using proteins annotated as ATIs, serpins and WGA for each variety, first the LFQ values of each protein were standardized according to the Eq. (3):

3 $$y_{{{\text{ij}}}} = \, \left( {x_{{{\text{ij}}}} {-}x_{{.{\text{j}}}} } \right) \, /s_{{\text{j}}} ,$$

where *x*_ij_ is the *i*th varietal value for the *j*th trait (protein), *x*_.j_ and *s*_j_ are the mean and standard deviation of the *j*th trait. Then, the standardised values of each protein for the respective variety were summed. We assigned equal weights to all proteins that were used to calculate the allergen index (Table 1).

#### Hierarchical clustering

For hierarchical clustering the data was scaled, and Euclidian distance was calculated. Hierarchical clustering was performed using “hclust” function of the statistical software R^34^ by implementing Ward’s agglomeration method^38^.

## Supplementary information

Supplementary Table S1.

Supplementary Table S2.

Supplementary Table S3.

Supplementary Table S4.

Supplementary Table S5.
